# A Facile Method for Generating a Smooth and Tubular Vessel Lumen Using a Viscous Fingering Pattern in a Microfluidic Device

**DOI:** 10.3389/fbioe.2022.877480

**Published:** 2022-05-02

**Authors:** Ting-Yuan Tu, Yen-Ping Shen, Sei-Hien Lim, Yang-Kao Wang

**Affiliations:** ^1^ Department of Biomedical Engineering, National Cheng Kung University, Tainan, Taiwan; ^2^ Medical Device Innovation Center, National Cheng Kung University, Tainan, Taiwan; ^3^ International Center for Wound Repair and Regeneration, National Cheng Kung University, Tainan, Taiwan; ^4^ AIM Biotech Pte Ltd., Singapore, Singapore; ^5^ Department of Cell Biology and Anatomy, College of Medicine, National Cheng Kung University, Tainan, Taiwan

**Keywords:** viscous fingering, HUVECs, permeability coefficient, microvasculature, microfluidics

## Abstract

Blood vessels are ubiquitous in the human body and play essential roles not only in the delivery of vital oxygen and nutrients but also in many disease implications and drug transportation. Although fabricating *in vitro* blood vessels has been greatly facilitated through various microfluidic organ-on-chip systems, most platforms that are used in the laboratories suffer from a series of laborious processes ranging from chip fabrication, optimization, and control of physiologic flows in micro-channels. These issues have thus limited the implementation of the technique to broader scientific communities that are not ready to fabricate microfluidic systems in-house. Therefore, we aimed to identify a commercially available microfluidic solution that supports user custom protocol developed for microvasculature-on-a-chip (MVOC). The custom protocol was validated to reliably form a smooth and functional blood vessel using a viscous fingering (VF) technique. Using VF technique, the unpolymerized collagen gel in the media channels was extruded by less viscous fluid through VF passive flow pumping, whereby the fluid volume at the inlet and outlet ports are different. The different diameters of hollow tubes produced by VF technique were carefully investigated by varying the ambient temperature, the pressure of the passive pump, the pre-polymerization time, and the concentration of collagen type I. Subsequently, culturing human umbilical vein endothelial cells inside the hollow structure to form blood vessels validated that the VF-created structure revealed a much greater permeability reduction than the vessel formed without VF patterns, highlighting that a more functional vessel tube can be formed in the proposed methodology. We believe the current protocol is timely and will offer new opportunities in the field of *in vitro* MVOC.

## Introduction

Blood vessels are ubiquitous and key components throughout the human body and are involved in many physio- and pathological processes (Dvorak et al., 1995; Dewhirst and Secomb, 2017). One of the fundamental roles of blood vessels in vascular functions is to maintain the vascular barrier to regulate proper biomolecular transport and tissue homeostasis. Dysregulation of such barrier function occurs in a variety of diseases and can lead to detrimental health conditions and prolonged treatment progression, such as edema, hemorrhage and chronic infection (Tsukita et al., 2001; Abbott et al., 2010). Consequently, advancing the understanding of the molecular mechanism regulating blood vessel permeability through experimental systems has emerged as an indispensable need for vascular biologists in the past few decades.

Several studies using *in vivo* experimental animals and *in vitro* cell cultures have shown insights into elucidating some of the mechanisms that contribute to the changes in the selective endothelial barrier functions (Mooradian et al., 2005; McRae et al., 2018). However, while the *in vivo* vascular microenvironment allows for the vessel to be studied under tissue physiochemical interactions, the complex *in vivo* nature not only impedes the vessel from being studied in a reductionist manner but also makes it difficult to control the changes in physiologic flow. Conversely, transwell assay is the most commonly applied *in vitro* assay to study barrier function (Katt and Shusta, 2020), but the inability to create physiological resemblance makes it difficult to recapitulate the *in vivo* architecture. These limitations have therefore instigated the need for a better experimental platform.

The immense progress of the microfluidic organ-on-a-chip technique has proven to be an enabling tool to better recapitulate various fundamental and applied vascular-related diseases and functions using microvasculature-on-a-chip (MVOC) (Park et al., 2013; Bai et al., 2015). Advances in these platforms have greatly facilitated a variety of vascularization strategies (Wang et al., 2018), which created an avenue easily permitting scientists to deepen the knowledge of many microvasculature-related biological functions and physical properties in the format of a microchip. To study endothelial barrier function in MVOCs, the most utilized approach would be to create a predesigned microchannel using polydimethylsiloxane (PDMS) or a hydrogel matrix followed by seeding endothelial cells (ECs) to form a monolayer along the inner wall of the channel (Montgomery et al., 2014; Tien, 2014; Polacheck et al., 2019). While these approaches have provided new insights into cell behaviors and function in many *in vitro* blood vessel studies, PDMS-based soft lithography has remained the main manual fabrication process followed by device formation through the creation of device inlets, outlets and enclosed microchannels. Moreover, each microfluidic vascular assay often requires establishing a unique operational workflow or protocol tailored for each chip design. For instance, one of the most commonly used EC lining-based methods may engage a series of maneuvers beginning from the removal of the microneedle or PDMS rod to form a hollow lumen, followed by a subsequent coating of extracellular matrix (ECM) to foster EC adhesions. Clearly, there is a lack of standardization in most of the lab-invented devices; translating the techniques from the bench into a routine end-user workflow will require increased robustness and throughput.

In contrast, a few commercially available microfluidic chips may offer a new alternative to conventional bench work development in the space of *in vitro* blood vessel studies (Mercurio et al., 2019; Ching et al., 2021). For instance, mass produced microfluidic chips through injection molding not only allow for minimum batch-to-batch variation but also permit production and sales at reasonable cost. The microfluidic chip product made from AIM Biotech is one example that facilitates 3D cell cultures for highly reproducible *in vitro* vessel and micro-vessel network studies (Son et al., 2019; Park et al., 2020; Zhao et al., 2021). The salient features of the AIM chip allow for incorporation of defined biochemical gradients, co-culture of different cell types and generation of accurate flow, enabling a more pathological and physiologically relevant microenvironment. In addition, the designs are also compatible with various imaging techniques including the conventional confocal microscopy. Therefore, visualization of the biological processes within the systems can be achieved with high optical resolution (Walji et al., 2021).

Viscous fingering (VF) technique is a facile method to create a continuous tubular lumen structure along a single microfluidic channel by displacing collagen through pumping less viscous fluid through without needing the insertion of an external mold (Bischel et al., 2012; de Graaf et al., 2019b). By seeding endothelial cells on the tubular collagen lumen surface, the blood vessel structure could then be attained and matured over time. The improvement of VF was further demonstrated by de Graaf et al., (2019)b who demonstrated that VF could be readily achieved *via* passive pumping using pressure-driven flow (PDF) (de Graaf et al., 2019), highlighting a simple technique to fulfill a facile workflow without requiring an additional pump and fluidic consumables. While passive pumping can be modulated *via* hydraulic pressure differences through a single fluidic channel, the AIM chip that contains two fluidic channels that are connected by a porous gel channel have not been validated with the use of VF.

In this work, we aim to explore whether VF can be integrated with a commercially available microfluidic chip to serve as a facile methodology for generating a smooth and tubular vessel lumen. Collagen type I was utilized and investigated under several factors affecting the VF lumen, including incubation temperature, collagen partial polymerization time (PPT), hydrostatic PDF, and collagen concentration (CC). Following the seeding of human umbilical vein endothelial cells (HUVECs) into the lumen, the formation of the three-dimensional (3D) vessel structure was assessed using confocal microscopy. Finally, the permeability of vessels formed either directly in the microchannel or with the VF-collagen lumen was measured. The results validated a reliable and straightforward method in commercial AIM chips for generating smooth and tubular vessel lumens with improved barrier function.

## Materials and Methods

### Cell Culture

HUVECs (Bioresource Collection and Research Center, Hsinchu, Taiwan) were used between passages 4 and 9 and maintained in EGM^®^-2 endothelial growth medium (CC-3162, Lonza, Basel, Switzerland) in T75 tissue culture flasks precoated with 50 µg ml^−1^ collagen coating solution for 30 min at 37°C to foster cell adhesion and growth (Park et al., 2013). The collagen coating solution was prepared using collagen type I (3.61 mg ml^−1^, Corning, NY, United States) and diluted in 0.02 N acetic acid (Sigma-Aldrich, MO, United States).

### Microfluidic Chip

**FIGURE 1:**
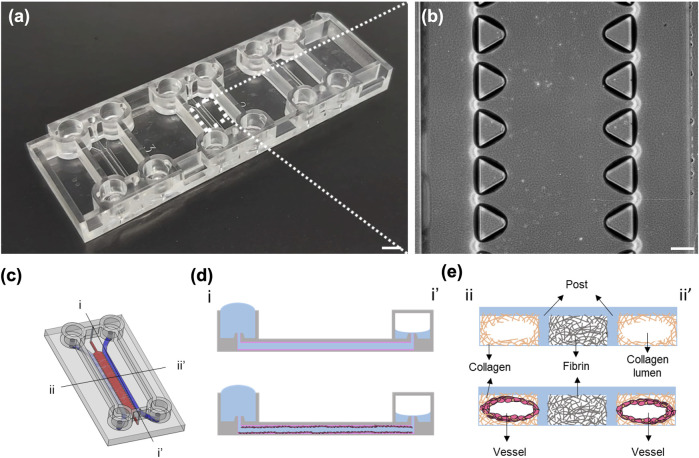
Images of the AIM chip and the schematic illustration of the viscous fingering (VF) processes. **(A)** Photograph of an AIM chip. Each chip comprises three independent experimental sites for three experiments. Scale bar: 5 mm. **(B)** Phase contrast image of the three channels and the trapezoidal micropillars that are designed to contain unpolymerized hydrogel within the hydrogel channel at each experimental site. Scale bar: 200 µm. **(C)** Schematic illustration of the experimental site in an AIM chip, with two media channels (blue) and one gel channel (red). **(D)** The cross-sectional view (i-i′) of the media channel depicting the formation of a lumen within the collagen using the VF technique and subsequent seeding of HUVECs in the lumen. **(E)** The cross-sectional view (ii-ii′) of the lumens within the collagen and vessel.

Commercially available microfluidic chips and chip holders (DAX-1 and HOL-2, AIM Biotech) were used for this study, as shown in Figure 1. The chip contains three independent microfluidic experimental sites, each with a footprint of 75 mm × 25 mm, allowing for the experiments to be performed in triplicate each time. Each experimental site on the chip contains three microfluidic channels, including two media channels on the side for cell culture and media replacement and one hydrogel channel in the middle. The hydrogel channel is separated from the media channels by two parallel arrays of trapezoidal posts containing unpolymerized hydrogel without spillage. Detailed information can be found on the official AIM Biotech website (https://www.aimbiotech.com/).

### Hydrogel Preparation

The fibrinogen working solution (6 mg ml^−1^) was prepared by dissolving 3 mg of fibrinogen powder (F8630, Sigma-Aldrich) in PBS in a 37°C water bath for 1 h. Lyophilized thrombin powder (T6634, 100 Unit, Sigma-Aldrich) was dissolved in 1 ml of sterile deionized water to make a thrombin stock solution. The thrombin stock solution was then added to PBS to prepare a thrombin working solution (4 unit ml^−1^). For the preparation of the fibrin gel mixture (3 mg ml^−1^), fibrinogen and thrombin working solutions were mixed in a 1:1 volume ratio in a microcentrifuge tube on ice. Collagen type I gel at concentrations of 2, 2.5 and 3 mg ml^−1^ and pH 7.4 was prepared by mixing 10 ×PBS (14416015, Corning) with phenol red 0.5 M NaOH solution, sterile deionized water, and rat-tail type I collagen (354236, Corning) in microcentrifuge tubes on ice.

### Device Operation and Collagen Lumen Verification

Given that several factors, including temperature, PDF, PPT and CC, are involved in the formation of the collagen lumen in the media channels using the VF technique, a detailed experimental workflow for this study is summarized (Supplementary Figure S1), and the detailed device operation for the formation of the collagen lumen is illustrated (Supplementary Figure S2). First, the fibrin gel mixture was injected into the gel channel and allowed to form a gel for 15 min at room temperature (RT) at 25°C. Each media inlet reservoir was then loaded with 15 µl of collagen gel with different CCs at RT. The collagen gel spontaneously flowed to the outlet of the media reservoirs due to a capillary force and cover the entire media channels. Each chip comprised three experimental sites (six media channels), and therefore, the operation could be repeated six times. In the event of incomplete collagen gel filling, where the entrance or exit was not fully covered (as depicted in Supplementary Figure S3), the chip needed to be tilted or the collagen needed to be extracted from the outlet reservoir by pipetting. For VF, hydrostatic PDF could be generated to purge unpolymerized collagen out of the outlet by changing different volumes of culture media at inlet and outlet reservoirs (Walker and Beebe, 2002). Appropriate PPT was determined when the collagen gel was drawn out from the microcentrifuge tube until the media was added to the media reservoir. After partial polymerization, the chip was then incubated at 37°C for 30 min to allow complete polymerization of the remaining type I collagen gel. For proper measurement and verification of the VF-formed collagen lumen, the chips were imaged using an inverted phase-contrast microscope (TS100-F, Nikon, Tokyo, Japan). Microbeads (0.1%, 5.0∼5.9 µm, SPHERO^TM^ polystyrene particles, Spherotech, Inc., Lake Forest, IL, United States) were diluted ten times in PBS and injected into the collagen lumen so that microbeads could attach along the surface of the collagen to make the lumen visible.

### Human Umbilical Vein Endothelial Cell Seeding in the Microfluidic Channels

The growth of HUVECs in the microfluidic channels was compared between the control (without VF) and experimental (with VF) groups. For the control group, media channels were first coated with fibronectin solution (33 µg ml^−1^, Sigma-Aldrich) in EGM-2 media and incubated at 37°C for 1 h. Channels were then washed twice by adding 70–50 µl (the volume added to the inlet and outlet reservoirs) of EGM-2. The procedures for seeding HUVECs within the chips were identical in both conditions (with/without VF). PDF was created by adding 70–50 µl (inlet-outlet) to the media channel reservoirs. Then, 10 µl of HUVEC suspension at a density of 3 million cells ml^−1^ was added into the media inlet reservoirs under incubation at 37°C for 15 min. To ensure that the entire surface of the collagen gel was covered by HUVECs, the seeding step was repeated four times. Each time, a 90° rotation of the chip perpendicular to the direction of the flow was performed. Media was changed daily, and HUVECs formed a lumen structure around the VF-formed collagen gel after 2 days of culture.

### Fluorescent Staining and Imaging

The HUVEC monolayer in AIM chips was stained using standard immunocytochemistry procedures. The staining reagents and washing buffer were perfused through media channels using 70–50 µl of PDF unless otherwise specified. First, the EC monolayer was washed with PBS three times. Then, the cells were fixed with 4% paraformaldehyde (Sigma-Aldrich) at RT for 15 min and washed three times with PBS. HUVECs were permeabilized with superblock blocking buffer (Thermo Fisher Scientific) containing 0.1% Triton X-100 (Sigma-Aldrich) for 60 min at RT. A primary antibody against human VE-cadherin (1:200; Ab33168, Abcam, Cambridge, MA, United States) diluted in PBS was added to the lumen and incubated at 4°C overnight. The next day, the cells were washed with PBS 3 times. Then, Alexa Fluor 488-conjugated secondary antibody (1:200; Abcam), phalloidin-TRITC (1:200; GeneTex, Irvine, CA, United States), and Hoechst (1:1,000; 33342, Thermo Fisher Scientific) were incubated for 1 h at RT. Finally, the device was washed with PBS five times. Fluorescence images were acquired by scanning confocal microscopy (FV3000, Olympus, Tokyo, Japan).

### Permeability Coefficient Measurement

Complete HUVEC lumen structures could be formed in the chips 3 days after cell seeding in both groups with/without VF. The permeability of the monolayer was assessed by perfusing 1 mg ml^−1^ of 70 kDa fluorescent dextran (FD70S, Sigma-Aldrich) solution into the HUVEC lumen side. Images were taken at 0, 5, 15, and 75 min by an inverted fluorescence microscope (IX83, Olympus). The average intensity values were extracted from each image by ImageJ, and the permeability coefficient (P_d_) was derived *via* the equation (Baxter et al., 1987; Huxley et al., 1987; Ho et al., 2017) shown as follows:

$$P_{d}=\frac{1}{t_{2}-t_{1}}\times \frac{I_{receiver}^{t2}-I_{receiver}^{t1}}{I_{donor}^{t1}-I_{receiver}^{t1}}\times \frac{V_{receiver}}{A_{barrier}}$$

where $I_{receiver}^{t1}$ is the intensity measured on the area that received dextran at time 1, $I_{receiver}^{t2}$ is the intensity measured on the same area at time 2, $I_{donor}^{t1}$ is the intensity measured on the area where donor dextran is present at time 1, $V_{receiver}$ is the volume (the area where $I_{receiver}^{t1}$ is measured multiplied by the height of the channel) that received dextran, and $A_{barrier}$ is the area of the boundary separating the receiver and donor.

### Statistical Analysis

All data are expressed as the mean ± standard deviation. The statistical significance was determined and compared at every position in the different channels by one-way ANOVA with GraphPad Prism 7.0 (GraphPad Prism Software Inc., San Diego, CA, United States). A *p*-value < 0.05 was considered significantly different, in which * represents *p* < 0.05, ** represents *p* < 0.01, and *** represents *p* < 0.001, and ****p < 0.0001.

## Results

Considering that each of the experimental sites of the AIM chip comprised two media channels at the sides and one gel channel in the center, the ECM formed inside the gel channel should be intact and provide a sufficient amount of physical support prior to lumen generation in the media channels under pressure-driven VF. Given that both fibrin and collagen are two essential ECM components and are commonly used materials for the *in vitro* engineering of blood vessels, we first investigated the collagen and fibrin gel introduced into the gel channel to test its stability and intactness upon injecting collagen into the media channels (refer to Supplementary Figure S4). The results showed that fibrin could provide better structural support with less deformability upon collagen injection. Therefore, fibrin was primarily used in the gel channel, and collagen was evaluated in the media channel in conjunction with the application of VF.

### The Effect of Extracellular Matrix, Temperature, and Partial Polymerization Time on Lumen Formation

**FIGURE 2:**
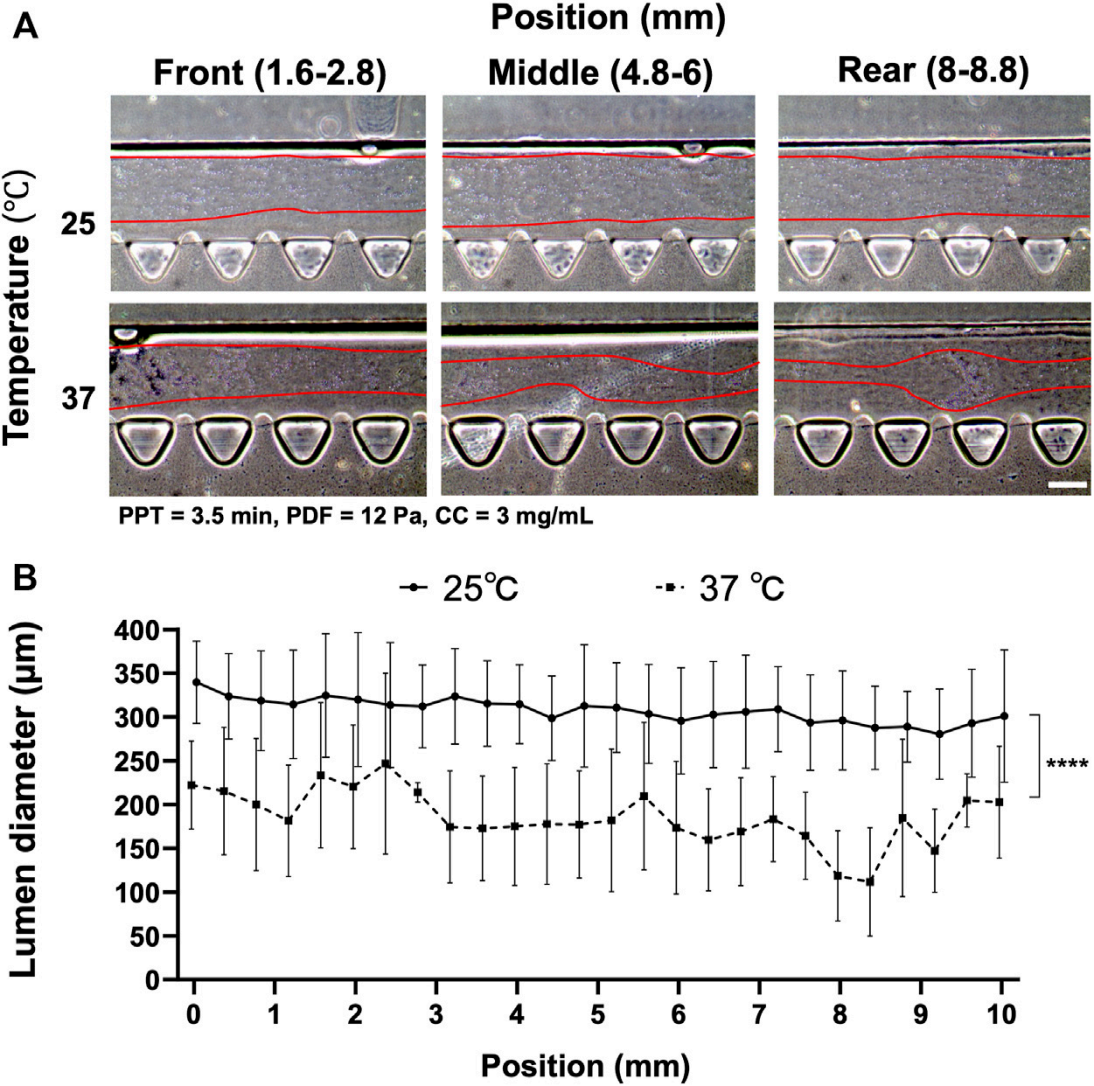
Comparison of the collagen lumen formed at 25°C or 37°C, where pressure-driven flow (PDF) applied under volume difference between inlet and outlet at 12 Pa, partial polymerization time (PPT) at 3.5 min, and collagen concentration (CC) at 3 mg ml^−1^. **(A)** Morphology of the collagen lumen at different locations of the media channel, i.e., front (1.6–2.8 mm), middle (4.8–6 mm), and back (8–8.8 mm) positions. **(B)** Quantification of the average collagen lumen diameter at different locations in the media channel pre-polymerized at RT and 37°C. Scale bar: 200 µm.

Collagen polymerization is dependent on the environmental temperature and time under neutral pH ∼7.4. The prerequisite of implementing VF requires only partially polymerized collagen so that unpolymerized ECM can be displaced by the less viscous fluid. Hence, the first experiment was set out to investigate a suitable temperature that was facile in operation and reproducible with VF. We compared the effect of temperature between 25°C and 37°C by fixing the rest of the parameters, i.e., PDF at 12 Pa, PPT at 3.5 min and CC at 3 mg ml^−1^ (Figure 2). Different locations of the media channel of the device (front, middle and rear position) were imaged to show the distribution of the patterned lumen, where the boundary of the collagen lumen was marked by the red lines (Figure 2A). Collagen lumen pre-polymerized at 25°C was found to be comparatively smoother than that at 37°C, which exhibited a larger fluctuation in lumen size distribution. The average diameters of the collagen lumen were measured every 0.4 mm across the entire length of the media channel, and the results showed that pre-polymerization at RT could lead to a larger lumen diameter that was approximately 300–330 μm, as compared to the120–250 µm diameter in the group that was pre-polymerization at 37°C (Figure 2B).

**FIGURE 3:**
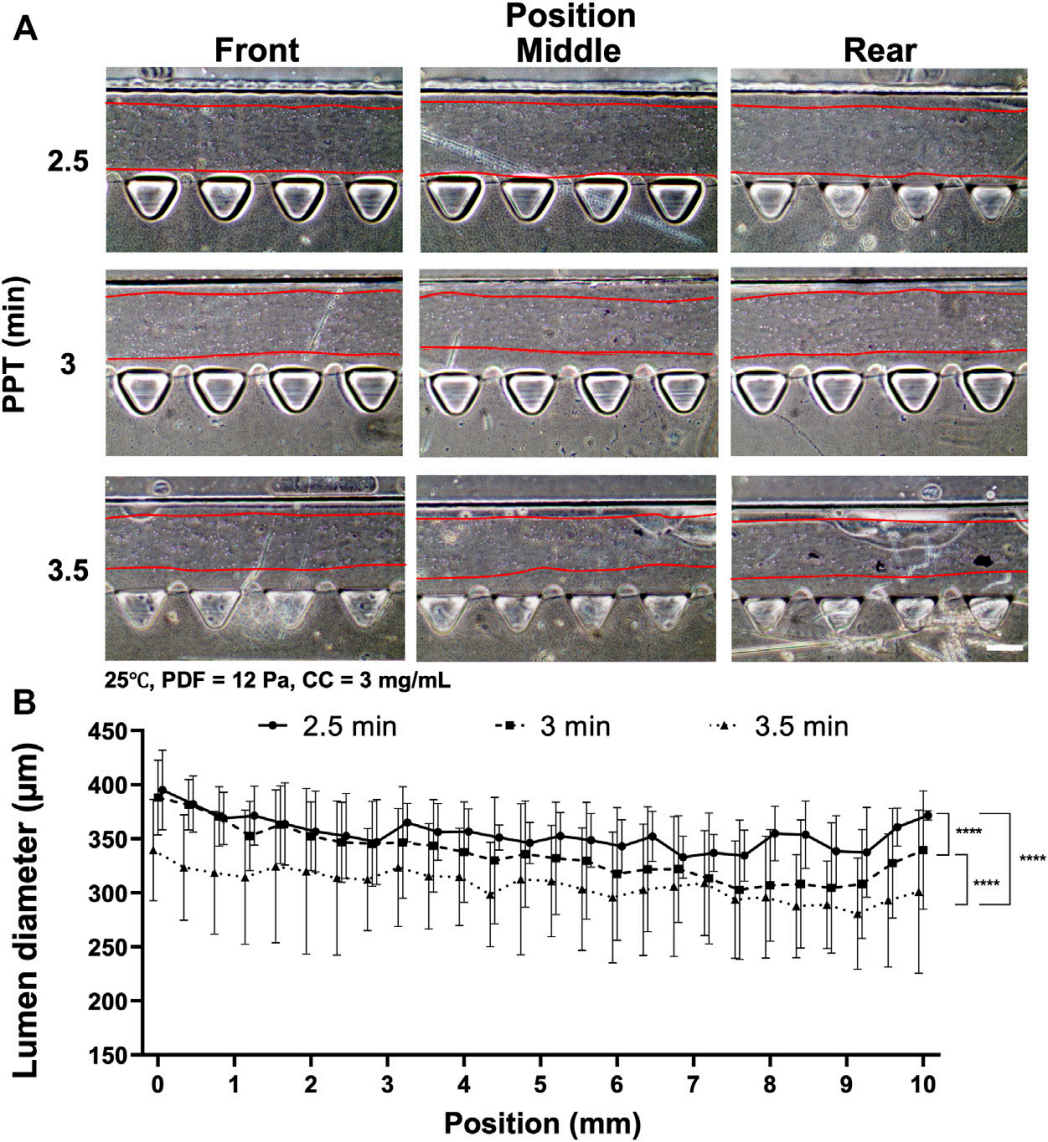
Comparison of the collagen lumen formed at different PPTs, i.e., 2.5, 3, and 3.5 min, where the temperature was set at 25°C, PDF at 90–50 µl, and CC at 3 mg ml^−1^. **(A)** Morphology of the collagen lumen formed at different locations of the media channel at different PPTs. **(B)** Quantification of the average collagen lumen diameter at different locations in the media channel polymerized at a PPT of 2.5, 3, or 3.5 min. Scale bar: 200 µm.

Another factor affecting collagen lumen diameter was associated with partial polymerization of the collagen incubated at different time points under the effect of VF. PPT was investigated at 2.5, 3 and 3.5 min, where the rest of the parameters were controlled at 25°C, PDF at 12 Pa and CC at 3 mg ml^−1^ (Figure 3). The images showed that a smooth lumen pattern could be observed under different PPTs across the front, middle and rear positions of the media channel (Figure 3A). Measurement of collagen lumen indicated that PPT at 2.5 min could yield the average lumen diameters from 350–390 μm, 3 min group distributed from 330–380 μm, and 3.5 min group distributed from 300–350 µm (Figure 3B). From the results, the sequence of the lumen diameter from wide to thin corresponded to the PPT condition 2.5, 3, 3.5 min.

### The Effect of Pressure-Driven Flow and Collagen Concentration on Collagen Lumen Formation

**FIGURE 4:**
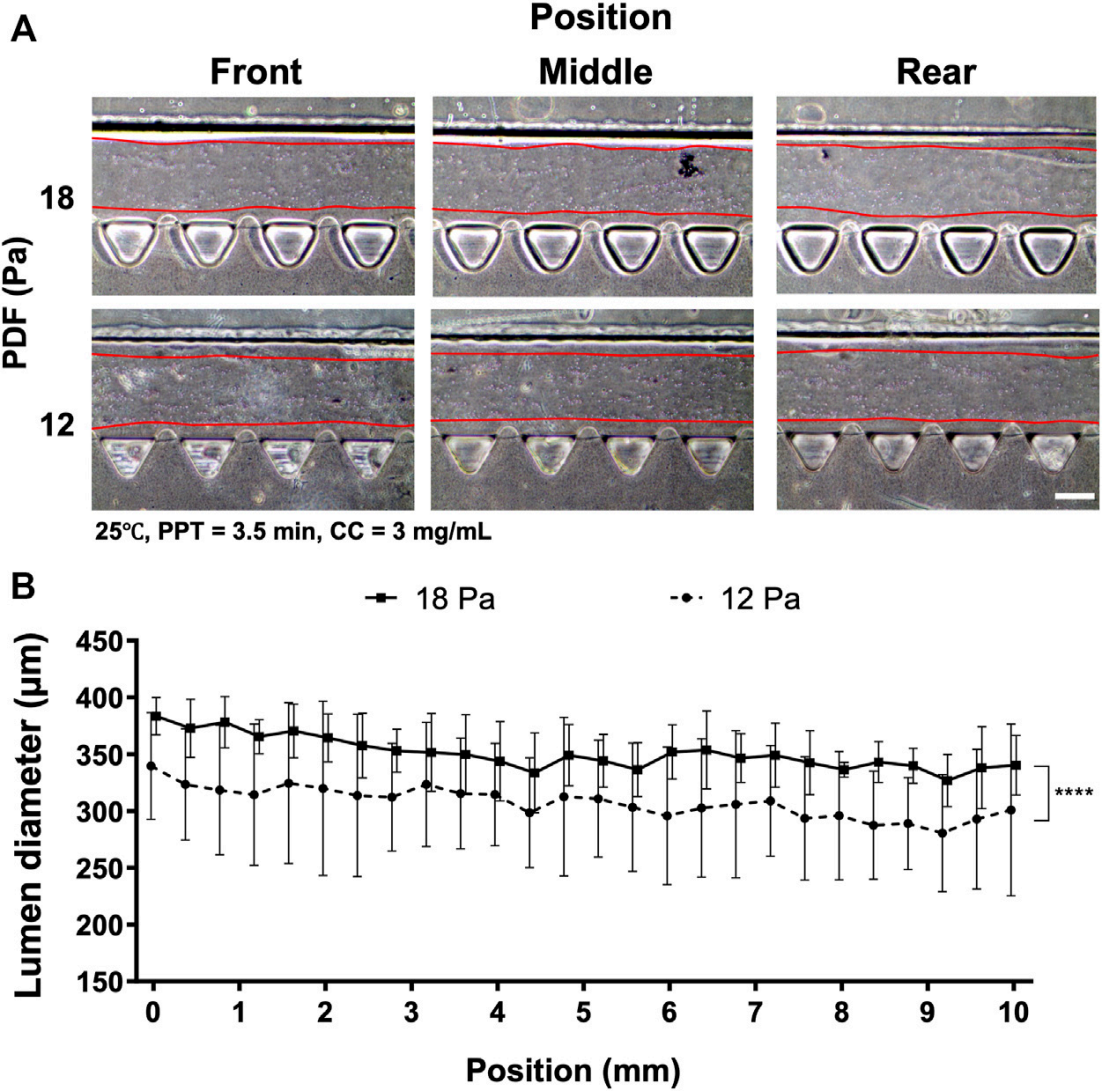
Comparison of the collagen lumen formed at different PDF values of 18 and 12 Pa, where the temperature was set at RT, PPT at 3.5 min and CC at 3 mg ml^−1^. **(A)** Morphology of the collagen lumen formed at different locations of the media channel. **(B)** Quantification of the average collagen lumen diameter at different locations in the media channel polymerized at PDFs of 18 and 12 Pa. Scale bar: 200 µm.

Given that VF-created patterns also largely depended on the levels of PDF, the PDF was then investigated at 18 and 12 Pa, respectively, where the remaining parameters were set at 25°C, PPT at 3.5 min and CC at 3 mg ml^−1^ (Figure 4). The images of collagen lumen distribution in the media channels under different PDFs are shown (Figure 4A). Measuring collagen lumen size revealed that PDF at 18 Pa could yield average lumen diameters from 350–380 µm wider than that of the 12 Pa group distributed from 300–350 µm (Figure 4B).

**FIGURE 5:**
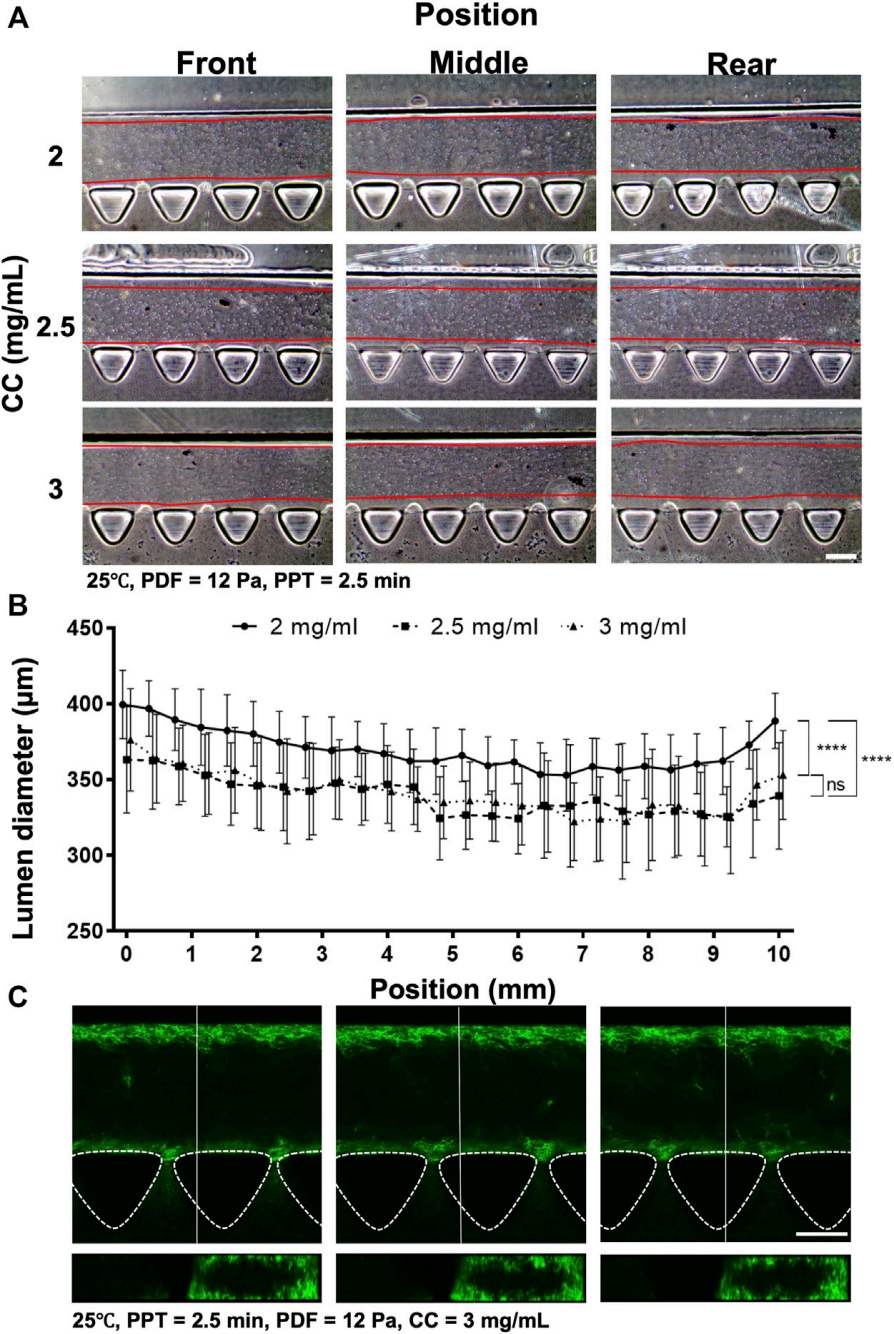
Comparison of the collagen lumen formed at different CCs at 2, 2.5, and 3 mg ml^−1^, where the temperature was set at 25°C, PDF at 12 Pa, and PPT at 2.5 min. **(A)** Morphology of the collagen lumen formed at different locations of the media channel at different CCs. **(B)** Quantification of the average collagen lumen diameter at different locations in the media channel. **(C)** Top- and cross-sectional views of the fluorescently labeled collagen lumen (3 mg ml^−1^). Scale bar: 200 µm.

The preliminary investigation suggested that PDF positively correlated with lumen size, yet PPT showed the inverse correlation. To reduce the waiting time during partial polymerization of the collagen, different CCs were investigated at 2, 2.5, and 3 mg ml^−1^ and PPT at 2.5 min at 25°C; the PDF was 12 Pa (Figure 5) and 15 Pa. Measurement of the collagen lumen indicated that CC at 2 mg ml^−1^ (*n* = 12) could yield average lumen diameters from 370–400 µm at different locations; the 2.5 mg ml^−1^ (*n* = 10) group, the average lumen diameters distributed from 330–360 μm, whereas in the 3 mg ml^−1^ (n = 16) group, the average lumen diameters distributed from 320–370 µm (Figures 5A,B). As increased CC is directly correlated with the higher stiffness and density of collagen, these results highlighted that different lumen diameters could be generated under different CCs and should be determined upon different expected applications. To visualize lumen formation, an Atto fluorescent-conjugated collagen (CC at 3 mg ml^−1^) labeled by Atto 488 NHS ester was patterned using the aforementioned patterned parameters with representative top and cross-section view images of the lumen at different locations (Figure 5C; Supplementary Figure S6). The results demonstrated consistent smooth and rounded collagen across the patterned media channel. The above results suggested that the CC also played an important role in affecting the diameter of the lumen. Given that there was no significant difference in lumen size between the 2.5 and 3 mg ml^−1^ groups, a further investigation was performed by raising the PDF to 15 Pa (refer to Supplementary Figure S5). The results showed that the average lumen diameter was 345, 360, and 378 μm, corresponding to CC at 2.0, 2.5 and 3 mg ml^−1^, respectively, which indicated that lumen size remained in a similar range for the same CC under different PDFs at 12 and 15 Pa. We also noticed that no PDF higher than 15 Pa should be applied, as the lumen within collagen would start to be destroyed by the increased stress from PDF.

### Formation of Vessels

**FIGURE 6:**
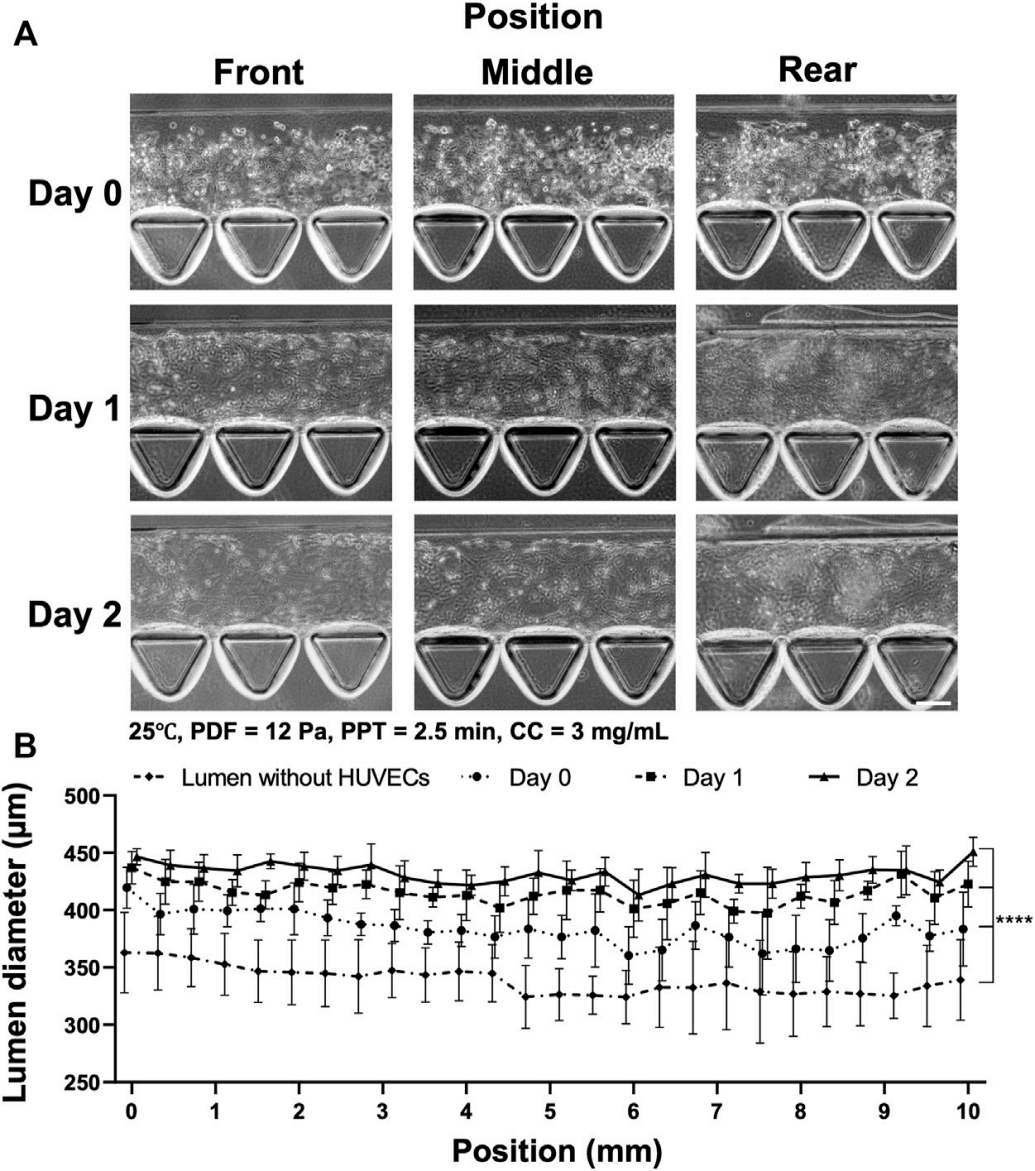
Process of HUVEC seeding and vessel formation in two days in the collagen lumen formed at 25°C, PDF at 12 Pa, PPT at 2.5 min, and CC at 3 mg ml^−1^. **(A)** Images of HUVEC seeding and vessel formation in 2 days at different locations of the device. Front (1.2–2 mm), middle (4.8–5.6 mm) and rear positions (8.4–9.2 mm) in the media channel. **(B)** Quantification of the average lumen diameter over time at different locations in the media channel. Scale bar: 100 µm.

Through the series of investigations, the collagen lumen was patterned at 25°C, PPT at 2.5 min, PDF at 12 Pa, CC at 3 mg ml^−1^, and HUVECs were seeded and cultured for 2 days to form a vessel-like structure in AIM chips (Figure 6). The images at the front, middle, and rear positions in the bright field showed the morphology of the cell seeding on Day 0, Day 1, and Day 2, respectively (Figure 6A). Measurement of vessels indicated that Day 0 (*n* = 12) yielded average lumen diameters from 330–420 µm at different locations. The Day 1 group was distributed from 420–440 μm, whereas the Day 2 group was distributed from 430–450 µm (Figure 6B). It was noted that culturing HUVECs that formed into the vessels degraded the ECM, which significantly enlarged the size of the collagen lumen, highlighting that firm structural support from the ECM is vital to maintain the integrity and size of the formed vessel. In fact, we also tested a sparser CC at 2.5 mg ml^−1^ that displayed compromised collagen without a clear lumen boundary at two days post-HUVEC seeding (data not shown). The validated condition was confirmed to yield a reproducible size of the collagen lumen and was also a facile operation for forming the vessels.

**FIGURE 7:**
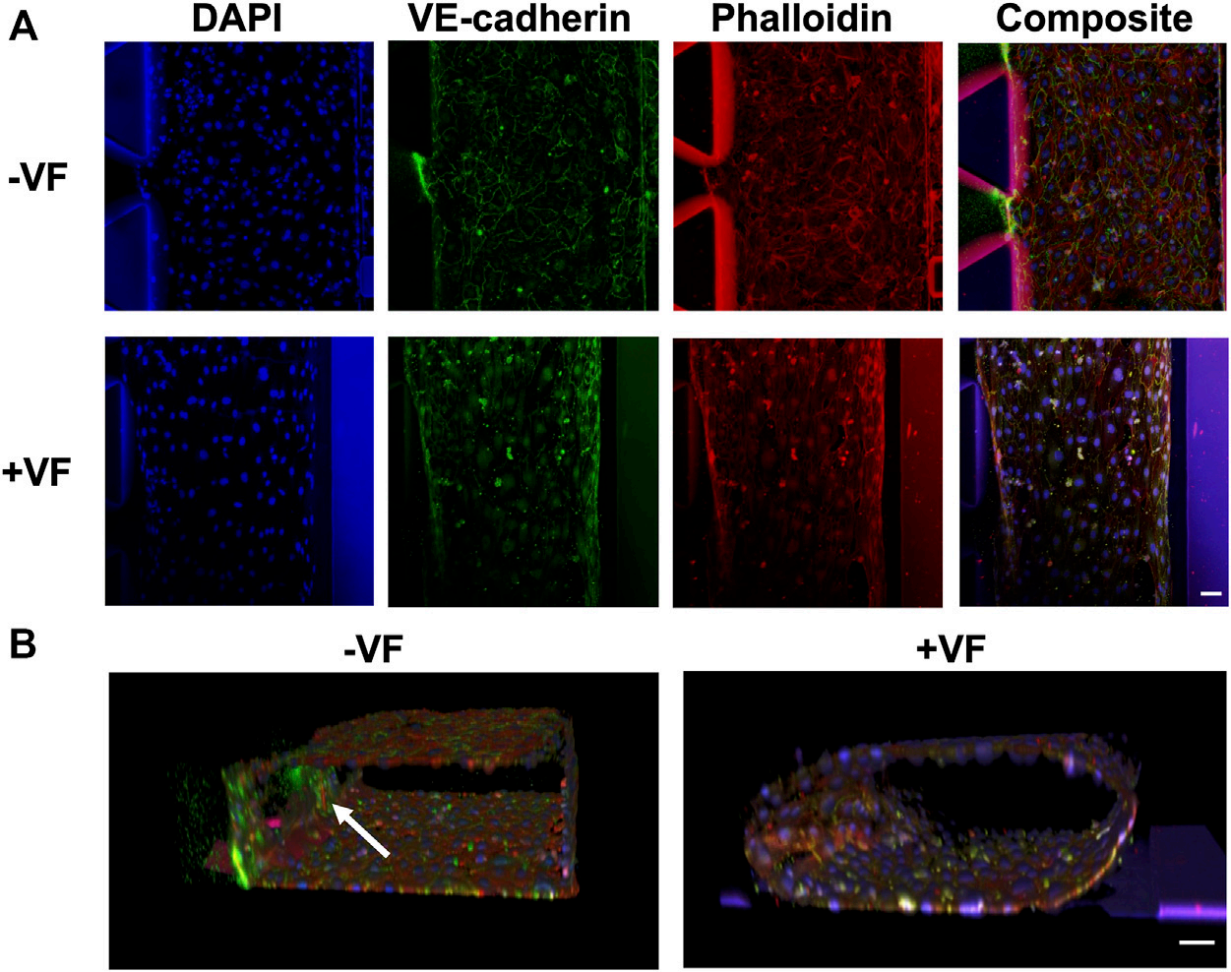
Immunofluorescence images of the vessels formed with or without the VF collagen lumen showing nuclear (blue), VE-cadherin (green) and F-actin (red). **(A)** Comparison of the vessels formed without the VF method (cells grown on the plastic surface of the AIM biochip) or with the VF method (cells grown on the collagen lumen). **(B)** 3D rendering of HUVEC vessels formed without or with VF collagen lumen. A white arrow indicates the cells that grow on the fibrin gel. Scale bar: 50 µm.

To investigate the morphology and structure of the vessel formed in the chip with or without VF, fluorescent staining was performed on nuclear (Hoechst), F-actin (phalloidin-TRITC) and VE-cadherin, as shown in Figure 7. The results showed that the growth of the HUVECs characterized in the previous experiments using bright field microscopy concurred with the proper formation of an enclosed vascular lumen, where the shape was defined by either the rectangular channel or tubular collagen lumen using a confocal microscope. While both groups demonstrated a compact and connected enclosed lumen, indicating the reproducible workflow for both procedures, the functional difference was unsure apart from their shapes and morphology. It was noted that the VE-cadherin stained HUVECs had a more homogenous fluorescent profile throughout the channel in the VF group while the fluorescent intensity of HUVECs markedly increased at the gel interfaces in the control group (as indicated by a white arrow).

### Diffusional Permeability Coefficient

**FIGURE 8:**
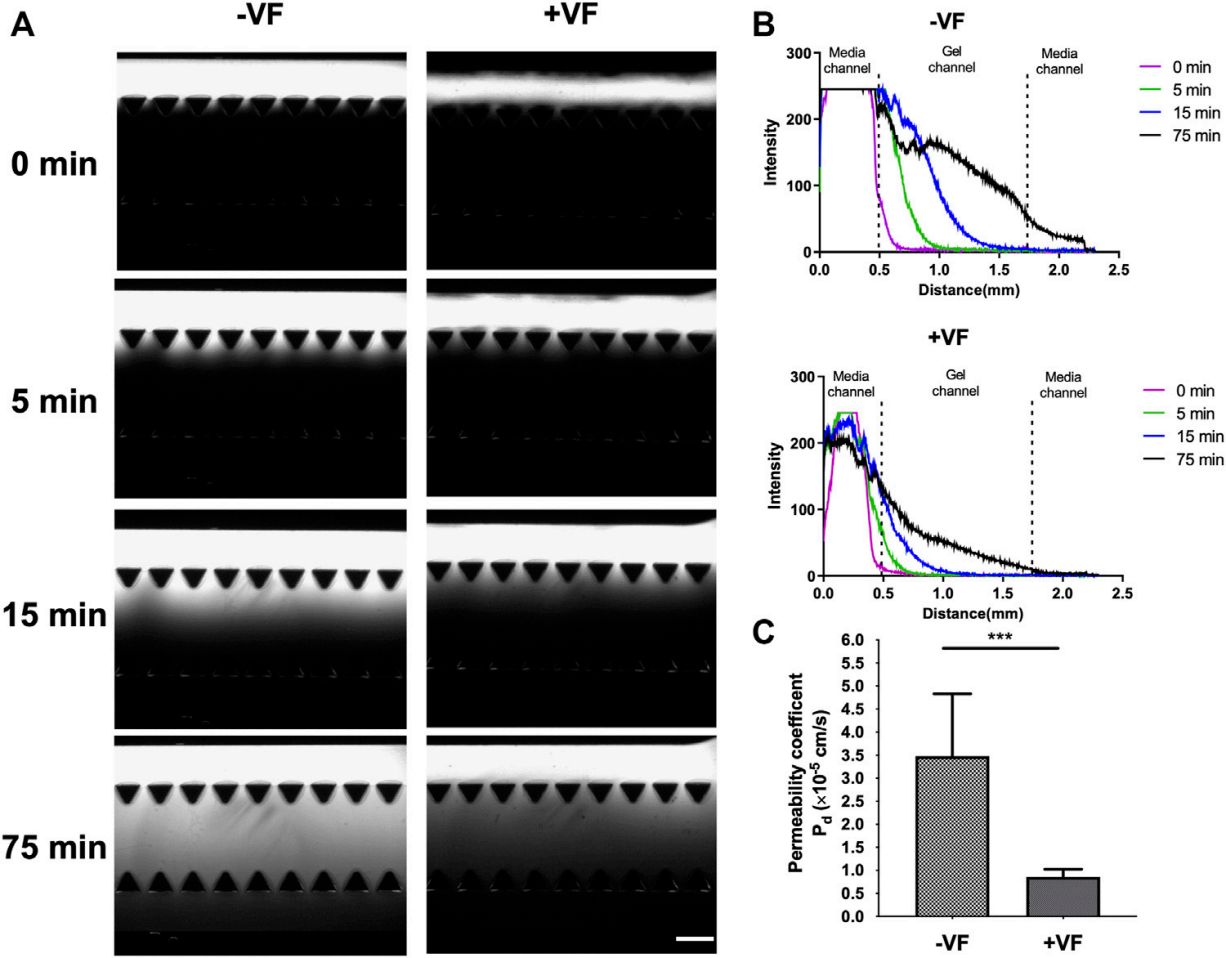
Measurement of permeability in vessels formed in the device without or with VF collagen lumen. **(A)** Fluorescent images of the 70 kDa FITC-dextran incubated in the lumen and observed at 0, 5, 15 and 75 min without or with VF collagen lumen. Scale bar: 500 µm. **(B)** Intensity maps of FITC-dextran observed without or with the VF collagen lumen at 0 (purple), 5 (green), 15 (blue) and 75 (black) min. **(C)** Permeability coefficient calculated from the fluorescent time-lapse images without (*n* = 4) and with the VF collagen lumen (*n* = 3).

The transport function of the HUVEC lumens can be quantitatively characterized *via* P_d_ using 70 kDa dextran and measured at time points 0, 5, 15, and 75 min, as shown in Figure 8. The fluorescent images revealed the trend of dextran diffusion in the two groups at each time point examined (Figure 8A). By quantifying the fluorescence intensity, the pattern of diffusion across different gel regions can be quantitatively determined (Figure 8B). The measurement showed that the average P_d_ was 3.48 (±1.35) × 10^–5^ cm s^−1^ in the control group (*n* = 4) and 8.61 (±1.53) ×10^–6^ cm s^−1^ in the VF group (*n* = 3). This result indicated a significant reduction in permeability by approximately an order of magnitude through growing vessels in the VF-formed collagen lumen than in the microfluidic channel wall, indicating that barrier function was better established by HUVECs through covering the gel-post interface with VF collagen.

## Discussion

Blood vessels play a significant role in various physiological and pathological responses of the human body. Various microfluidic MVOC platforms have been developed over the past decades by taking advantage of these microscale systems not only reducing the amount of working samples needed to save the precious primary cells and the expensive reagents but also creating spatial partitioning to compartmentalize cells and ECM through defined microstructures that are essential for the conducive growth of the microvasculature. In addition, the salient features of microfluidic platforms permit precise control of physical flow conditions, and the biochemical microenvironment also plays an important role in the maturation of *in vitro* vessels (van der Helm et al., 2016; Tsvirkun et al., 2017; Polacheck et al., 2019; Wang et al., 2022). However, most devices remain on the laboratory bench and fail to be more widely applied mainly due to a lack of standardization and throughput for end users. In this study, we proposed a reliable workflow for uniform smooth vessels formed *via* VF in conventional pillar-based 3D microfluidic chips. Multiple factors that are crucial in a day-to-day operation in performing VF in microfluidic chips were investigated, including environmental temperature, PDF, PPT and CC, which are all essential in forming the proper lumen.

Integrating ECM into the microfluidic device enables the microenvironment to have a better physiological resemblance than that of the standard flat surface culture on plasticware. Historically, coating of basement membrane proteins *via* laminin and fibronectin can foster EC adherence to the microchannel; on the other hand, mixing EC with fibril gel or collagen type I along with proper stimuli would facilitate MVOC through vasculogenesis and angiogenesis (Wang et al., 2018). Compared with the rapid and stable fibrin gelation through fibrinogen/thrombin interaction at RT, collagen fibrillogenesis was initiated through pH neutralization, and dependence on temperature and concentration became more apparent (Doyle, 2016). Our findings validated that RT can be a conducive temperature for partial polymerization under VF in AIM chips, as patterning the collagen lumen at RT eliminates the need for transportation of chips between the biological safety cabinet and incubator and can thus be more reproducible. It should be noted that the RT condition was established in the air-conditioned lab environment at approximately 25°C, and deviation from this temperature may have a significant impact on the formation of the collagen lumen and should be investigated separately with care.

One major difference between the AIM chip and other previous studies lies in the number of microfluidic channels involved during the VF process. The AIM chip contains two parallel media channels connected by a porous hydrogel channel in between. During the process of VF, the Saffman–Taylor instability evolves into the finger pattern that forms at the interface between two fluids in a porous medium (Homsy, 1987), which results in a variance in the VF lumen. The pressure difference established by PDF could also generate interstitial fluid flow across the hydrogel. The quality of the lumen structure formed in such microchannel design has not been studied under the VF. Therefore, we first change the PDF by adjusting volumes at the inlets and outlets in generating the VF. The hydrostatic pressure difference of 18 and 12 Pa corresponds to the volume ratio (inlet vs. outlet) at 90 µl vs. 30 µl and 90 µl vs. 50 µl. The results showed that lumen width dropped from 380 µm (76%) to 300 µm (60%) in the 500 µm width channel, which corresponded to Bischel et al. (2012) who reported that the lumen width decreases from approximately 80%–60% of the channel under the VF Kang et al. (2004) also demonstrated simulation results that the VF width was 75.4% of the channel width when the viscosity ratio was 10. Given that the width of the lumen within collagen was affected by the PDF, we also investigated the effect of the same PDF at 12 Pa, with different volumes (µl) in inlet and outlet reservoirs equal to 90–50, 80–40, and 70–30 ratios, for collagen lumen formation (refer to supplementary information and Supplementary Figure S7). A similar trend was observed in groups 90–50 and 80–40 µl. However, in 70–30 μl, the average lumen diameter became thinner than the others due to the minimum operable volume at 30 μl, which was designed in the AIM chip media reservoirs and could just cover the inlet and outlet. As 30 µl approaches the lower limit of the volume in media reservoirs, the success rate accounted for 33% of the total devices tested. It was noted that a smaller PDF could also be adjusted for lumens with smaller diameters, and the condition must be investigated further.

The EC lining-based method has been a major avenue for studies of endothelium function, such as blood brain barriers and cancer cell extravasation. Ho et al. (2017) demonstrated that microvessels could be formed to probe vascular permeability of nanoparticles in nanomedicine applications by seeding primary HUVECs in a pillar-based design microfluidic device similar to the AIM chip. The permeability coefficient of those vessels is 2.47 × 10^–5^ cm s^−1^ for a larger-sized molecule, 70 kDa dextran. However, molecular leakiness due to cell attachment was incomplete at the post side and formed vessels with discontinuous surfaces. Though complete endothelial monolayer can be formed in the media channels of AIM chips, there are variations of fluorescent intensity along the vessel where the VE-cadherin signal is stronger at the gel interfaces as compared to the post interfaces. In contrast, the VF approach could generate a smooth collagen lumen so that the entire surface in the media channel can be covered by collagen. This modification allowed the formation of a homogenous endothelium that led to the lower variability in permeability measurements and a lower permeability coefficient. Using VF, Bischel et al., (2013) created 3D endothelial-lined microvessels in a tubeless microfluidic for angiogenesis assays. While the study could achieve a low permeability of the microvessels at 4.73 (±0.75) × 10^–6^ cm s^−1^characterized by perfusing 40 kDa dextran through the vessels, simultaneous operation of multiple pumps and laborious processes in multi-layer soft lithography were inevitable to fabricate a microfluidic structure with different heights. Contrarily, the current reported VF method driven by hydrostatic PDF not only ensures that the operation is handy without involving added equipment or consumables but also guarantees an improved barrier function. Most importantly, the commercially available nature of the AIM chip enables the ease of accessibility and a high reproducibility which will be more conducive for biologists and medical researchers.

While the present study validated a reliable workflow to form smooth endothelium-lined MVOC with a VF lumen in multiple microfluidic channels in the commercial AIM chip, there are some limitations that should be taken into account. First, the vessels present an elliptical shape due to the rectangular design of the microfluidic channel, and the size of the vessels is also limited to ∼75% of the channel dimension under VF. To form a circular vessel with a smaller diameter, the channel dimension must be adjusted into a square with a corresponding size adjustment. Second, although the permeability coefficient of 8.61 (±1.53) × 10^–6^ cm s^−1^ achieved in VF lumen is comparable to other studies that were determined *in vitro*, the reported value is still higher than that of the permeability investigated under a perfusion of physiologic flow of 0.5–1 × 10^–6^ cm/s (Polacheck et al., 2019) and *in vivo* of 0.01–0.04 × 10^–6^ cm/s in the dermal microvasculature (Polacheck et al., 2017), which highlights the importance of incorporating biophysical cues and tissue specific architectures *in vivo* to reinforce the microvascular functions may be separately investigated. Third, the current reported work only maintained the microvessels for 4 days. Thus, more experiments are needed to investigate the possibility of long-term maintenance of vessels in the chips.

Despite these limitations, this study successfully validated a reliable and straightforward method in commercial AIM chips for generating smooth and tubular vessel lumens with improved barrier function. A user custom VF protocol was validated to reliably form a smooth and functional blood vessel. The VF patterns achieved at the temperature of 25°C had more homogenous diameter throughout and it was easier to operate than at 37°C. The collagen lumen diameter increased as the PDF increased; and increased as the PPT and the gel concentration decreased. The MVOC was achieved through the seeding of HUVEC onto the collagen lumen and it was further characterized by immunofluorescence. Most importantly, the lumen created by VF techniques allowed the formation of a endothelium that has tighter barrier function as measured by the permeability coefficient of fluorescent dextran. We believe the current protocol is timely and will offer new opportunities in the field of *in vitro* MVOC.

## Data Availability

The original contributions presented in the study are included in the article/Supplementary Material, further inquiries can be directed to the corresponding author.

## References

[B1] Abbott N. J. Patabendige A. A. K. Dolman D. E. M. Yusof S. R. Begley D. J. (2010). Structure and Function of the Blood-Brain Barrier. Neurobiol. Dis. 37, 13–25. 10.1016/j.nbd.2009.07.030 19664713

[B2] Bai J. Tu T.-Y. Kim C. Thiery J. P. Kamm R. D. (2015). Identification of Drugs as Single Agents or in Combination to Prevent Carcinoma Dissemination in a Microfluidic 3D Environment. Oncotarget 6, 36603–36614. 10.18632/oncotarget.5464 26474384 PMC4742198

[B3] Baxter L. T. Jain R. K. Svensjö E. (1987). Vascular Permeability and Interstitial Diffusion of Macromolecules in the Hamster Cheek Pouch: Effects of Vasoactive Drugs. Microvasc. Res. 34, 336–348. 10.1016/0026-2862(87)90066-5 2448593

[B4] Bischel L. L. Lee S.-H. Beebe D. J. (2012). A Practical Method for Patterning Lumens through ECM Hydrogels via Viscous Finger Patterning. J. Lab. Autom. 17, 96–103. 10.1177/2211068211426694 22357560 PMC3397721

[B5] Bischel L. L. Young E. W. K. Mader B. R. Beebe D. J. (2013). Tubeless Microfluidic Angiogenesis Assay with Three-Dimensional Endothelial-Lined Microvessels. Biomaterials 34, 1471–1477. 10.1016/j.biomaterials.2012.11.005 23191982 PMC3529167

[B6] Ching T. Toh Y.-C. Hashimoto M. Zhang Y. S. (2021). Bridging the Academia-To-Industry Gap: Organ-On-A-Chip Platforms for Safety and Toxicology Assessment. Trends Pharmacol. Sci. 42, 715–728. 10.1016/j.tips.2021.05.007 34187693 PMC8364498

[B7] de Graaf M. N. S. Cochrane A. van den Hil F. E. Buijsman W. van der Meer A. D. van den Berg A. (2019). Scalable Microphysiological System to Model Three-Dimensional Blood Vessels. APL Bioeng. 3, 026105. 10.1063/1.5090986 31263797 PMC6588522

[B8] Dewhirst M. W. Secomb T. W. (2017). Transport of Drugs from Blood Vessels to Tumour Tissue. Nat. Rev. Cancer 17, 738–750. 10.1038/nrc.2017.93 29123246 PMC6371795

[B9] Doyle A. D. (2016). Generation of 3D Collagen Gels with Controlled Diverse Architectures. Curr. Protoc. Cel Biol. 72, 10.20.1–10.20.16. 10.1002/cpcb.9 PMC5030718 27580704

[B10] Dvorak H. F. Brown L. F. Detmar M. Dvorak A. M. (1995). Vascular Permeability Factor/Vascular Endothelial Growth Factor, Microvascular Hyperpermeability, and Angiogenesis. Am. J. Pathol. 146, 1029–1039. 7538264 PMC1869291

[B11] Ho Y. T. Adriani G. Beyer S. Nhan P.-T. Kamm R. D. Kah J. C. Y. (2017). A Facile Method to Probe the Vascular Permeability of Nanoparticles in Nanomedicine Applications. Sci. Rep. 7, 1–13. 10.1038/s41598-017-00750-3 28386096 PMC5429672

[B12] Homsy G. M. (1987). Viscous Fingering in Porous Media. Annu. Rev. Fluid Mech. 19, 271–311. 10.1146/annurev.fl.19.010187.001415

[B13] Huxley V. H. Curry F. E. Adamson R. H. (1987). Quantitative Fluorescence Microscopy on Single Capillaries: Alpha-Lactalbumin Transport. Am. J. Physiology-Heart Circulatory Physiol. 252, H188–H197. 10.1152/ajpheart.1987.252.1.h188 3492924

[B14] Kang Q. Zhang D. Chen S. (2004). Immiscible Displacement in a Channel: Simulations of Fingering in Two Dimensions. Adv. Water Resour. 27, 13–22. 10.1016/j.advwatres.2003.10.002

[B15] Katt M. E. Shusta E. v. (2020). *In Vitro* Models of the Blood-Brain Barrier: Building in Physiological Complexity. Curr. Opin. Chem. Eng. 30, 42–52. 10.1016/j.coche.2020.07.002 32905326 PMC7469950

[B16] McRae M. LaFratta L. M. Nguyen B. M. Paris J. J. Hauser K. F. Conway D. E. (2018). Characterization of Cell-Cell junction Changes Associated with the Formation of a Strong Endothelial Barrier. Tissue Barriers 6, e1405774. 10.1080/21688370.2017.1405774 29388870 PMC5823545

[B17] Mercurio A. Sharples L. Corbo F. Franchini C. Vacca A. Catalano A. (2019). Phthalimide Derivative Shows Anti-angiogenic Activity in a 3D Microfluidic Model and No Teratogenicity in Zebrafish Embryos. Front. Pharmacol. 10, 349. 10.3389/fphar.2019.00349 31057399 PMC6479179

[B18] Montgomery M. Zhang B. Radisic M. (2014). Cardiac Tissue Vascularization: From Angiogenesis to Microfluidic Blood Vessels. J. Cardiovasc. Pharmacol. Ther. 19, 382–393. 10.1177/1074248414528576 24764132

[B19] Mooradian A. D. Haas M. J. Batejko O. Hovsepyan M. Feman S. S. (2005). Statins Ameliorate Endothelial Barrier Permeability Changes in the Cerebral Tissue of Streptozotocin-Induced Diabetic Rats. Diabetes 54, 2977–2982. 10.2337/diabetes.54.10.2977 16186401

[B20] Park J. H. Kim M. J. Kim W. J. Kwon K.-D. Ha K.-T. Choi B. T. (2020). Isolinderalactone Suppresses Human Glioblastoma Growth and Angiogenic Activity in 3D Microfluidic Chip and *In Vivo* Mouse Models. Cancer Lett. 478, 71–81. 10.1016/j.canlet.2020.03.009 32173479

[B21] Park Y. Tu T. Lim S. Clement I. (2013). *In Vitro* Microvessel Growth and Remodeling within a Three-Dimensional Microfluidic Environment. Cell Mol 7 (1), 15–25. 10.1007/s12195-013-0315-6 PMC3960002 24660039

[B22] Polacheck W. J. Kutys M. L. Tefft J. B. Chen C. S. (2019). Microfabricated Blood Vessels for Modeling the Vascular Transport Barrier. Nat. Protoc. 14, 1425–1454. 10.1038/s41596-019-0144-8 30953042 PMC7046311

[B23] Polacheck W. J. Kutys M. L. Yang J. Eyckmans J. Wu Y. Vasavada H. (2017). A Non-Canonical Notch Complex Regulates Adherens Junctions and Vascular Barrier Function. Nature 552, 258–262. 10.1038/nature24998 29160307 PMC5730479

[B24] Son S. Cho M. Lee J. (2019). Crumbs Proteins Regulate Layered Retinal Vascular Development Required for Vision. Biochem. Biophysical Res. Commun. 521, 939–946. 10.1016/j.bbrc.2019.11.013 31718797

[B25] Tien J. (2014). Microfluidic Approaches for Engineering Vasculature. Curr. Opin. Chem. Eng. 3, 36–41. 10.1016/j.coche.2013.10.006

[B26] Tsukita S. Furuse M. Itoh M. (2001). Multifunctional Strands in Tight Junctions. Nat. Rev. Mol. Cel Biol 2, 285–293. 10.1038/35067088 11283726

[B27] Tsvirkun D. Grichine A. Duperray A. Misbah C. Bureau L. (2017). Microvasculature on a Chip: Study of the Endothelial Surface Layer and the Flow Structure of Red Blood Cells. Sci. Rep. 7, 45036. 10.1038/srep45036 28338083 PMC5364477

[B28] van der Helm M. W. van der Meer A. D. Eijkel J. C. T. van den Berg A. Segerink L. I. (2016). Microfluidic Organ-On-Chip Technology for Blood-Brain Barrier Research. Tissue Barriers 4, e1142493. 10.1080/21688370.2016.1142493 27141422 PMC4836466

[B29] Walji N. Kheiri S. Young E. W. K. (2021). Angiogenic Sprouting Dynamics Mediated by Endothelial‐Fibroblast Interactions in Microfluidic Systems. Adv. Biol. 5, 2101080. 10.1002/adbi.202101080 34655165

[B30] Walker G. M. Beebe D. J. (2002). A Passive Pumping Method for Microfluidic Devices. Lab. Chip 2, 131–134. 10.1039/b204381e 15100822

[B31] Wang L.-C. Chang L.-C. Su G.-L. Chang P.-Y. Hsu H.-F. Lee C.-L. (2022). Chemical Structure and Shape Enhance MR Imaging-Guided X-ray Therapy Following Marginative Delivery. ACS Appl. Mater. Inter. 14, 13056–13069. 10.1021/acsami.1c24991 35253424

[B32] Wang X. Sun Q. Pei J. (2018). Microfluidic-based 3D Engineered Microvascular Networks and Their Applications in Vascularized Microtumor Models. Micromachines 9, 493. 10.3390/mi9100493 PMC6215090 30424426

[B33] Zhao X. Seah I. Xue K. Wong W. Tan Q. S. W. Ma X. (2021). Antiangiogenic Nanomicelles for the Topical Delivery of Aflibercept to Treat Retinal Neovascular Disease. Adv. Mater., 2108360. 10.1002/adma.202108360 34726299

