# Supplementary material for: A Facile Method for Generating a Smooth and Tubular Vessel Lumen Using a Viscous Fingering Pattern in a Microfluidic Device
Source: Front Bioeng Biotechnol. 2022 May 2;10:877480. doi: 10.3389/fbioe.2022.877480 (PMC9108369; doi:10.3389/fbioe.2022.877480)
Supplement: Supplementary file 1 [file DataSheet1.PDF]

frontiers

Supplementary Material

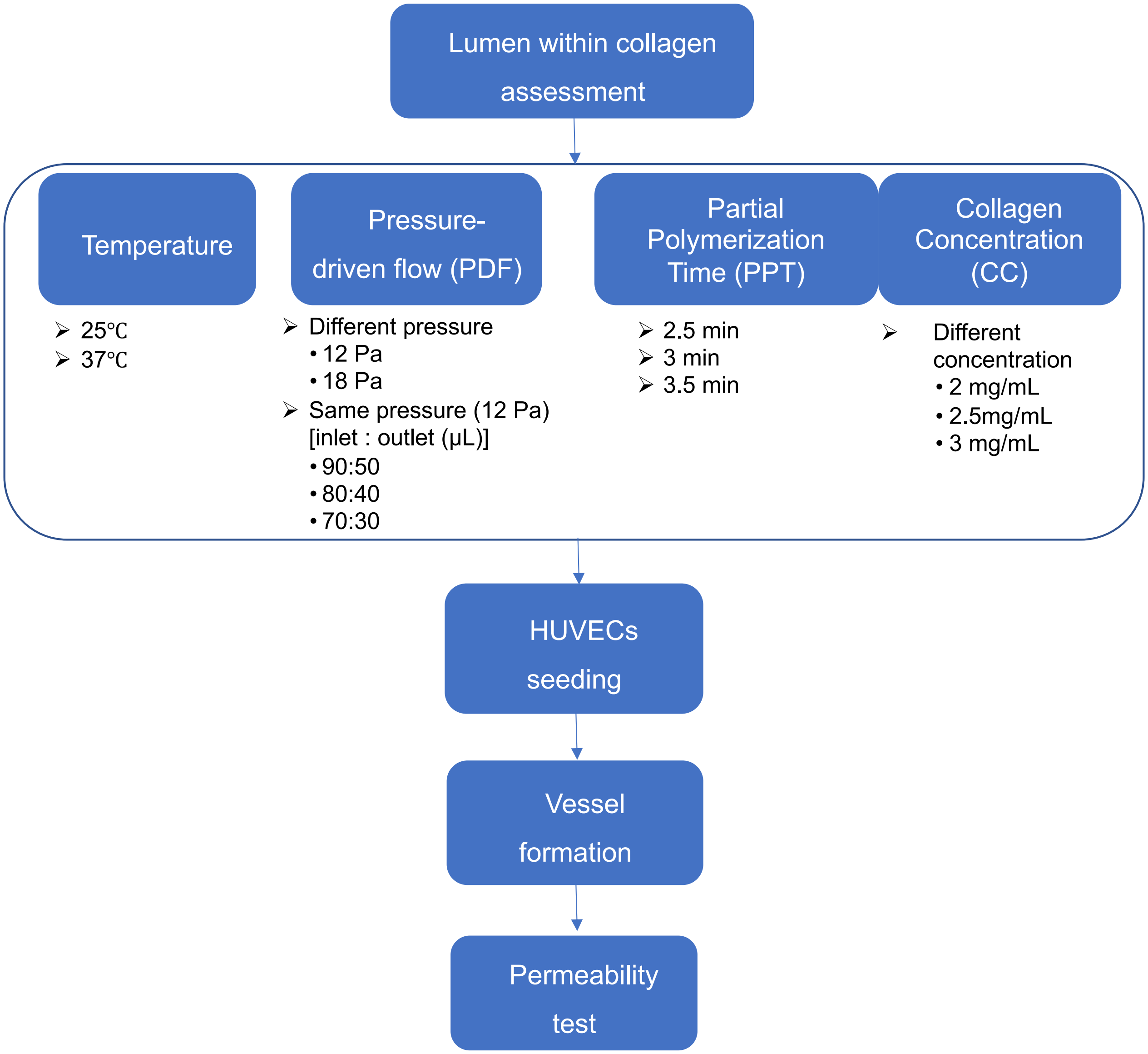

Figure S1. Experimental workflow and parameters involved in forming collagen lumen and microvasculature-on-chips (MVOC).

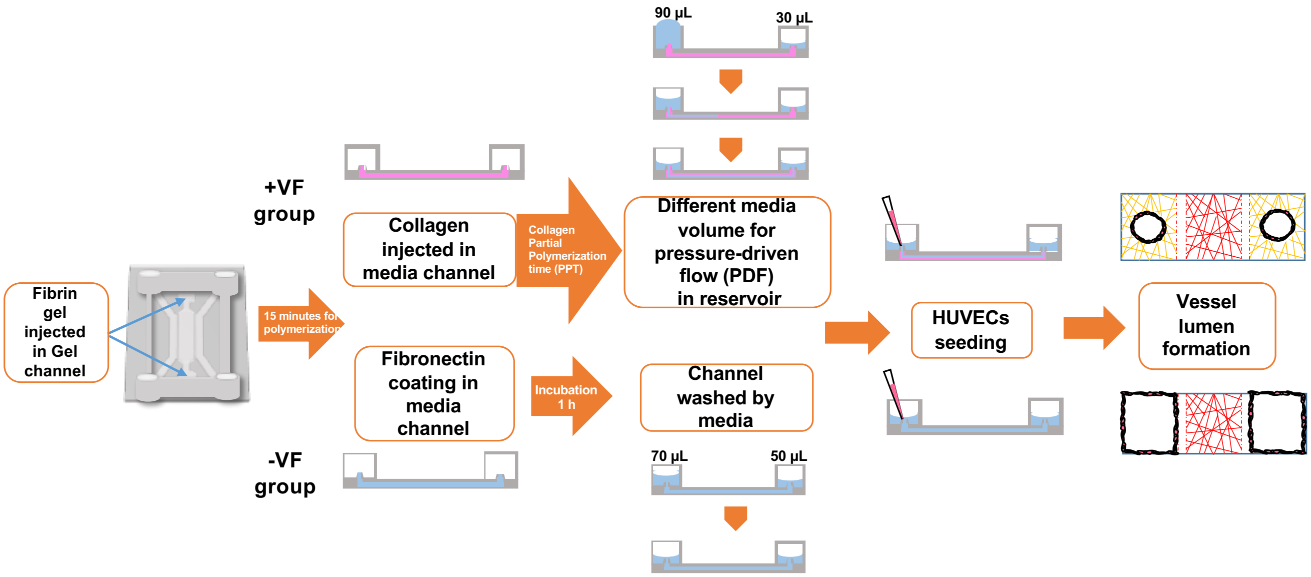

Figure S2. Device operation for the collagen lumen structure and control.

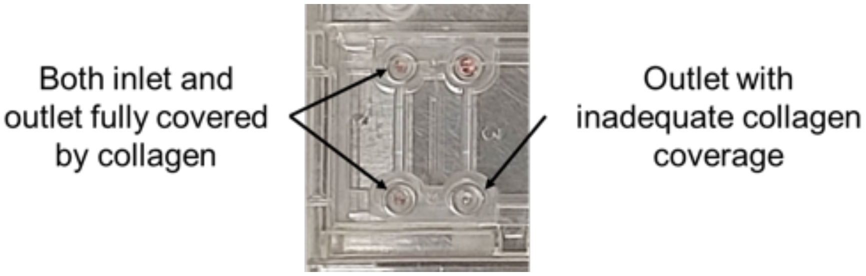

Figure S3. Collagen thoroughly covered both inlet and outlet (left) and inadequate coverage of the collagen at the outlet (right).

Identification of hydrogel in gel channel

Both type I collagen and fibrin gels were examined by adding them to the gel channel for

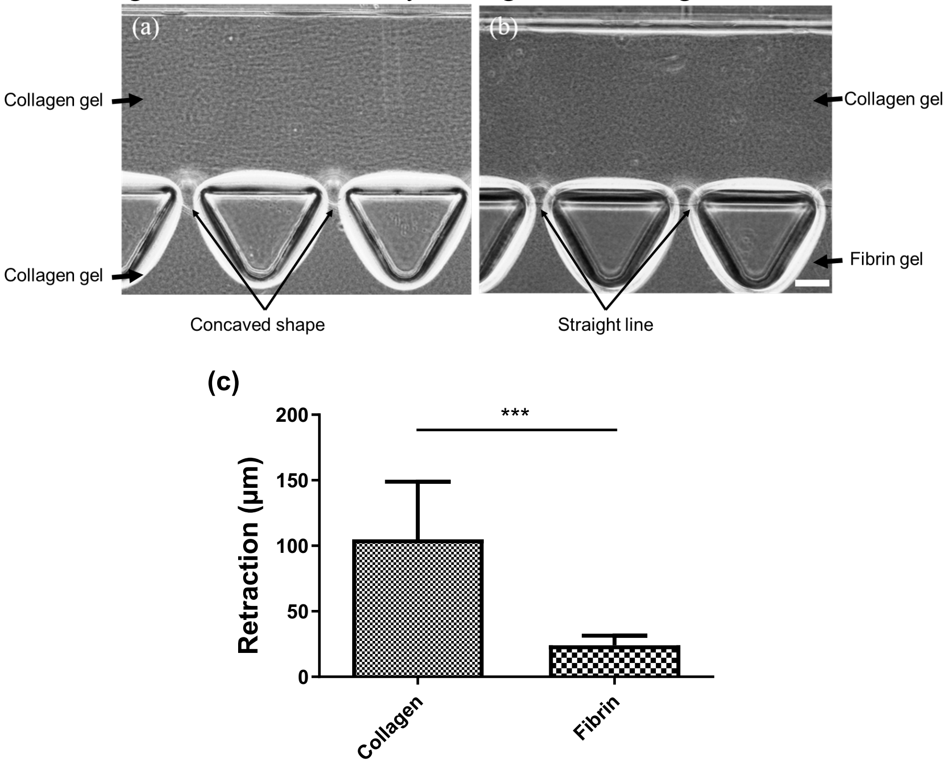

preliminary investigation (

Figure S4). The deformation of collagen gel between the gaps of the trapezoidal posts was observed to form a concaved shape after collagen injection at medial channels (

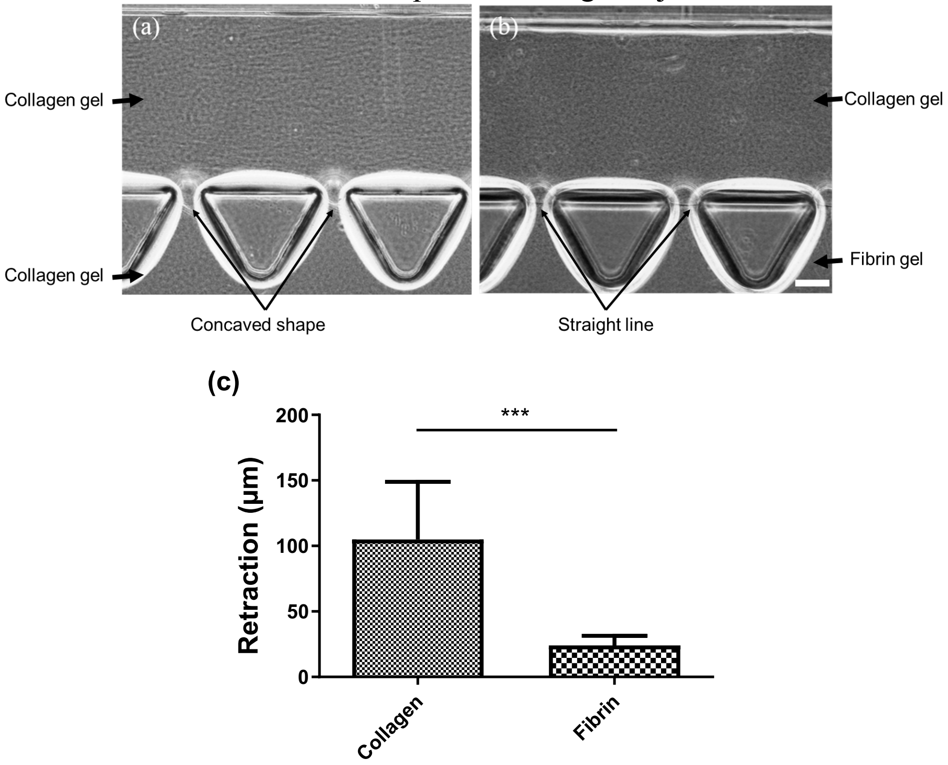

Figure S4a). The concaved gel region was retracted from the posts by about 105 (± 43.0) µm, suggesting the boundary of the collagen in the gel channel could be easily affected upon the injection of another collagen in media channels. Contrarily, replacing the collagen with fibrin gel has exhibited

improved gel containment between the gel posts (

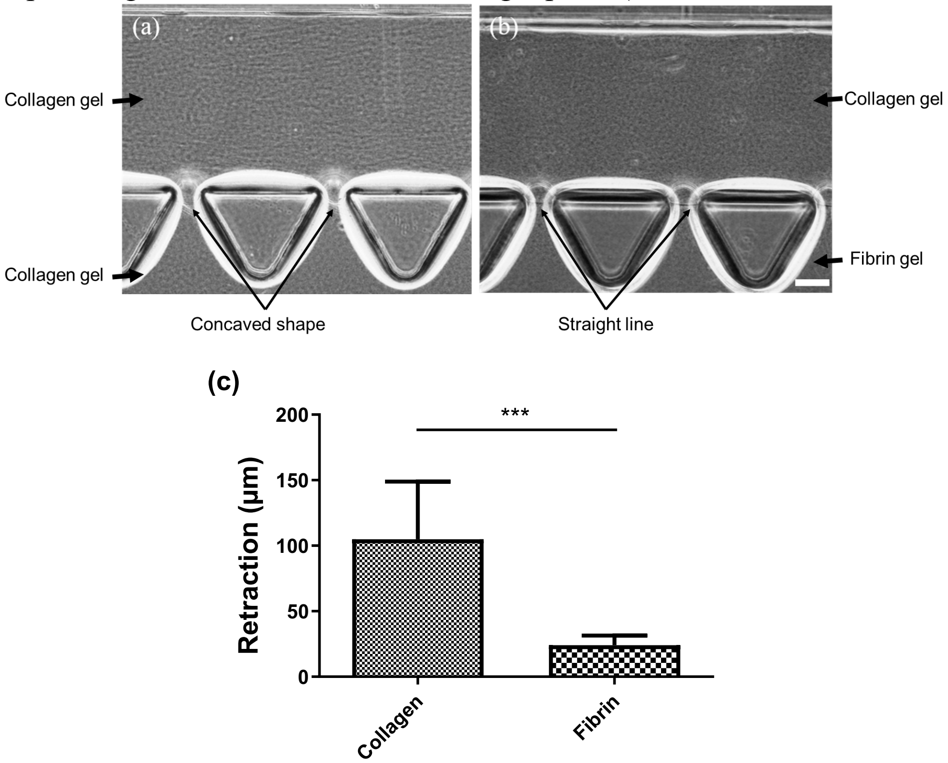

Figure S4b), where the boundary of fibrin gel remained nearly unretracted and formed a straight line and retracted from the posts by about 24 (± 7.1) μm between the gaps of the posts, highlighting the uniformity of gel boundary was more controlled in fibrin gel than that of the

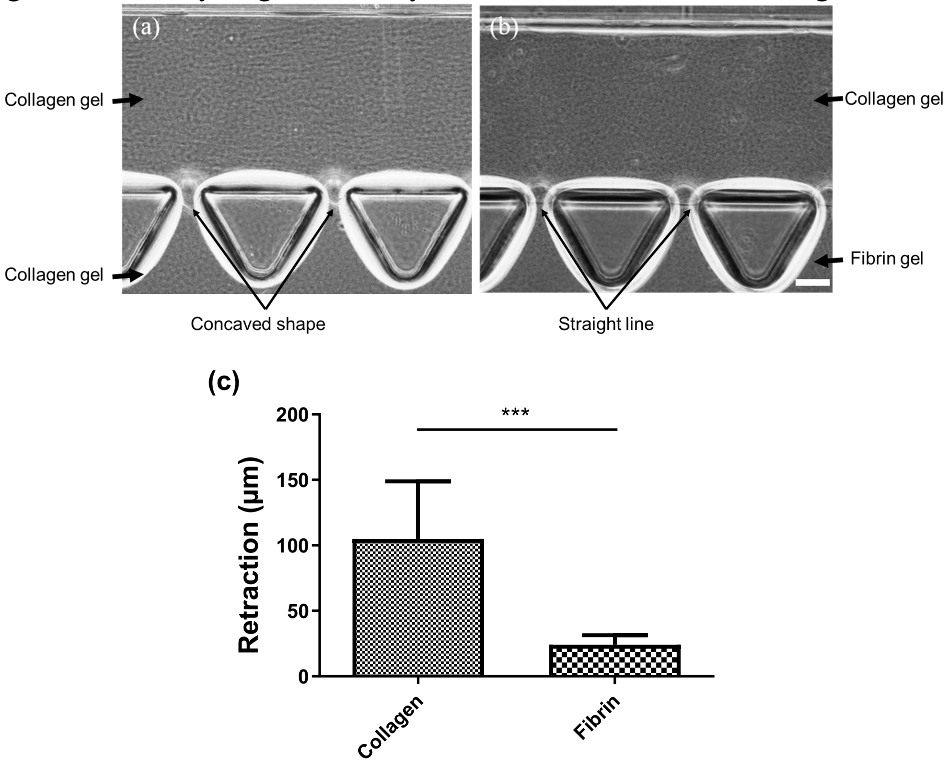

collagen (

Figure S4c). Therefore, fibrin was chosen as the base gel seeded in the gel channel. Otherwise, the gel in the gel channel got deformed and generated a concave shape between the posts. In

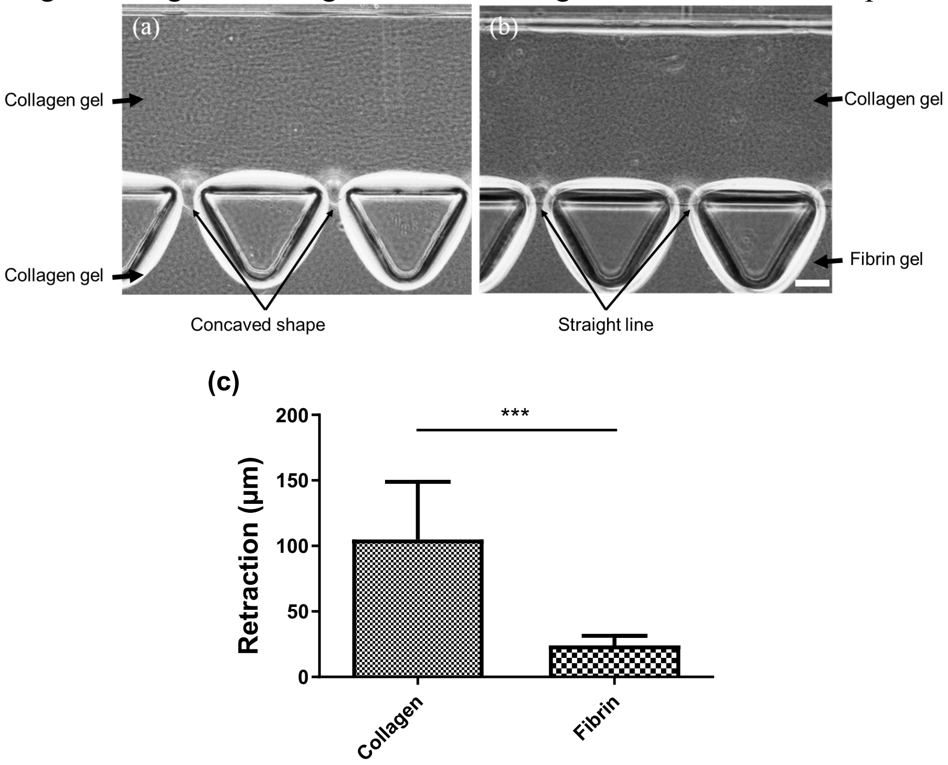

Figure S4, comparing the collagen, the deformation distance of collagen was 81 μm more than fibrin gel, and fibrin is more homogeneous than collagen.

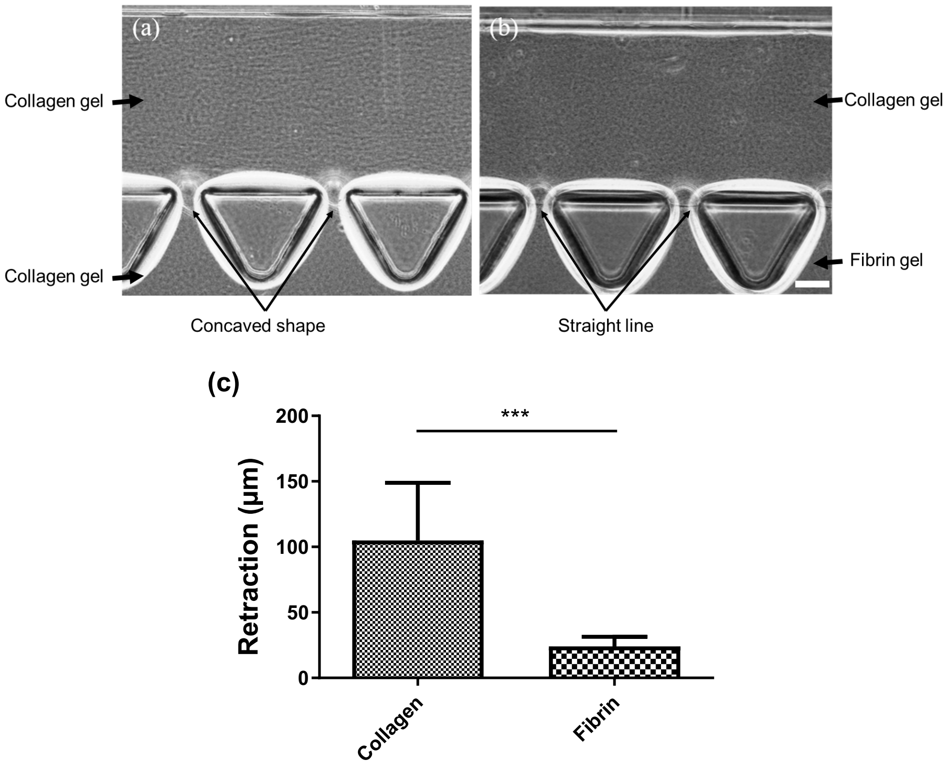

Figure S4. Uniformity of gel boundary between the gel channel posts after injection of collagen gel into the media channel. (a)collagen gel. (b)fibrin gel. (c)The comparison chart of the collagen and fibrin gel. Scale bar:100 µm

The effect of CC for collagen lumen formation when PDF at 15 Pa condition

To further clarify the effect of the CC for collagen lumen diameter, the collagen lumen was patterned at 25°C, PDF at 15 Pa, PT at 2.5 min, and CC at 2, 2.5, and 3mg mL^{-1} (Figure S5). The images of collagen lumen distribution in the media channels under different CC were presented (Figure S5a). Measurement of collagen lumen indicated that CC at 2 mg mL^{-1} could yield the average lumen diameters from 370—400 µm at a different location, 2.5 mg mL^{-1} group distributed from 360—380 µm, and 3 mg mL^{-1} group distributed from 340—370 µm (Figure S5b).

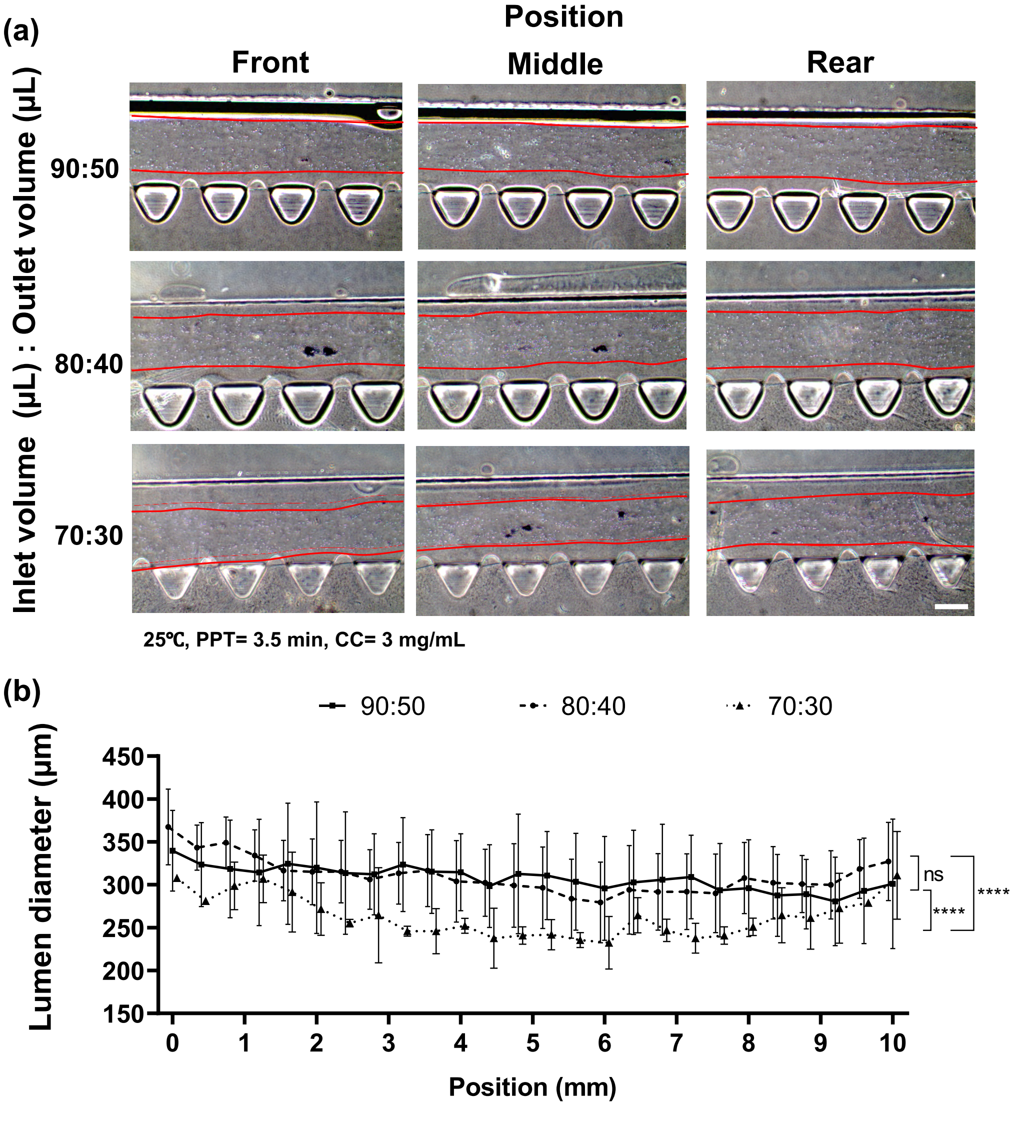

Figure S5. Comparison of the lumen within collagen formed at CC, i.e., 2, 2.5, and 3 mg mL^{-1}, where the temperature set at 25°C, PDF at 15 Pa, PT at 3.5 min. (a) Morphology of the collagen lumen formed at different locations of the media channel. (b) Quantification of the average collagen lumen diameter at different locations in media channel polymerized at different CC at 2, 2.5, and 3 mg mL^{-1}. Scale bar:200 µm

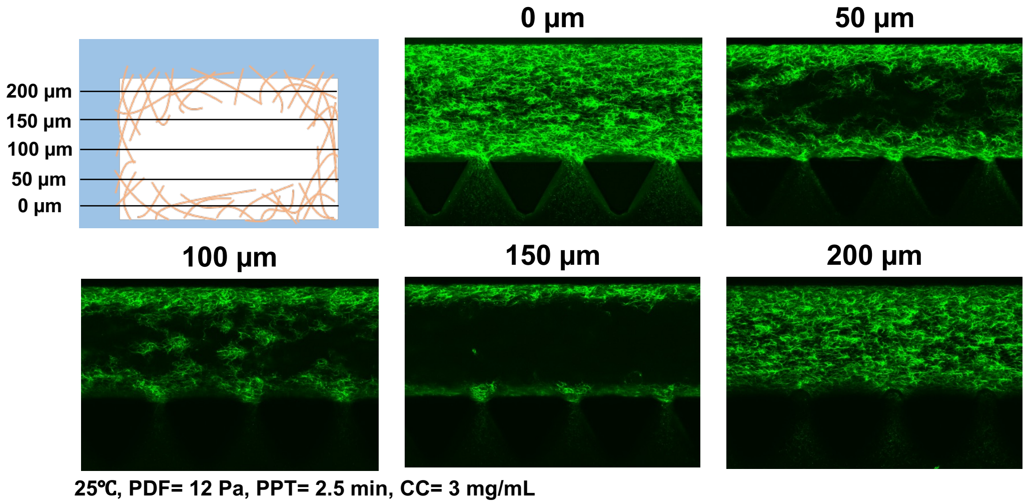

Figure S6. The image of fluorescent collagen in different 5 Z-layer (0, 50, 100, 150, 200 µm).

VF under the same pressure difference

To make sure the influence of pressure, first, the lumen was patterned at different volumes but under the same PDF. All conditions were patterned at 25°C, PT at 3.5 min, and CC at 3 mg mL^{-1}. The PDF were investigated with different volumes at inlet: outlet reservoirs (μL) 90:50, 80:40, and 70:30 (Figure S7). The images of collagen lumen distribution in the media channels under different media volumes were (Figure S7a). Measurement of collagen lumen indicated that PDF at 90:50 group could yield the average lumen diameters from 310—340 μm at a different location, 80:40 group distributed from 310—360 μm, whereas 70:30 group distributed from 240—310 μm (Figure S7b).

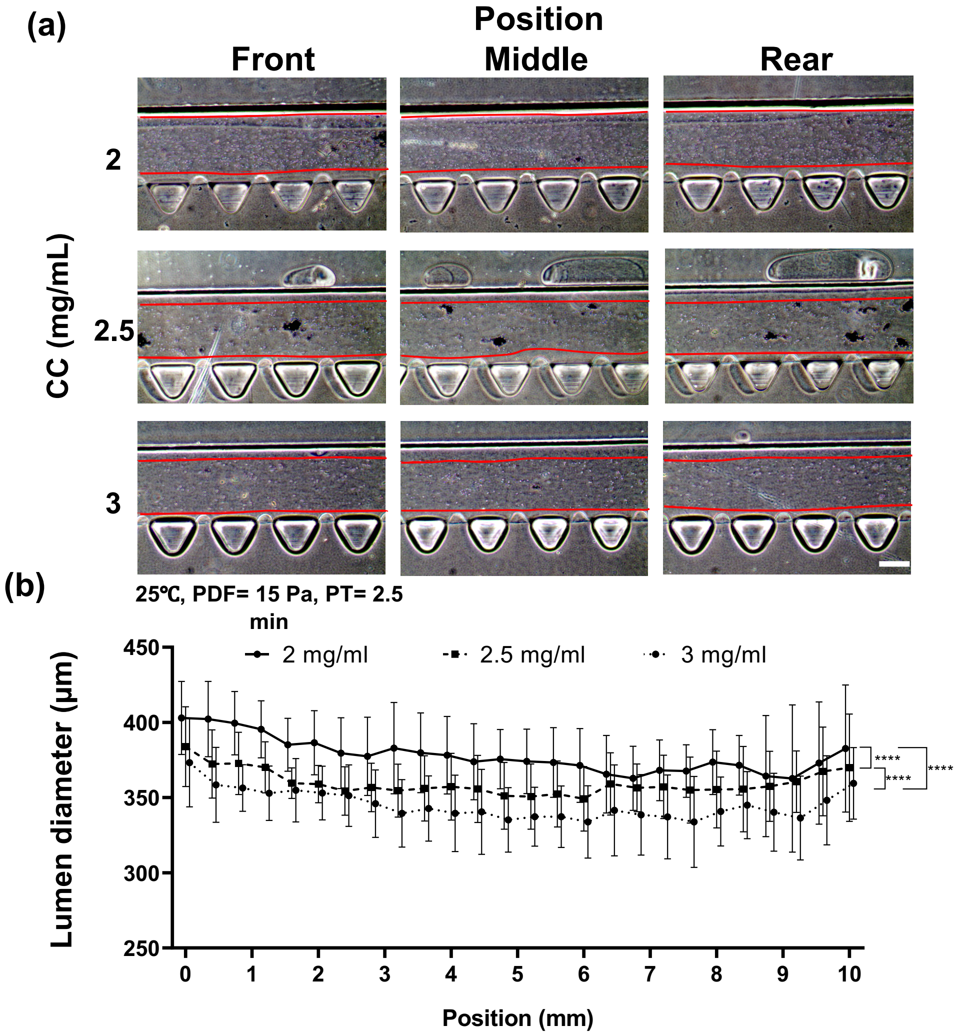

Figure S7. Comparison of the collagen lumen formed at same PDF, 12 Pa but different volume, i.e. (inlet ports: outlet ports(μl)) 90:50, 80:40, and 70:30 (12 Pa), where the temperature set at 25°C, PT at 3.5 min, and CC at 3 mg mL^{-1}. (a) Morphology of the collagen lumen formed at different locations of the media channel at different volumes (b) Quantification of the average collagen lumen diameter at different locations in media channel polymerized at PDF of 90:50, 80:40, 70:30. Scale bar:200 μm.
